# Total and individual PBC-40 scores are reliable for the assessment of health-related quality of life in Greek patients with primary biliary cholangitis

**DOI:** 10.2478/jtim-2023-0098

**Published:** 2023-09-02

**Authors:** Eirini I. Rigopoulou, Marianna Bakarozi, Ioannis Dimas, Konstantinos Galanis, Vasiliki Lygoura, Nikolaos K. Gatselis, Mairi Koulentaki, George N. Dalekos

**Affiliations:** Department of Medicine and Research Laboratory of Internal Medicine, National Expertise Center of Greece in Autoimmune Liver Diseases, General University Hospital of Larissa, Larissa 41110, Greece; European Reference Network on Hepatological Diseases (ERN RARE-LIVER), General University Hospital of Larissa, Larissa 41110, Greece; Hepatology Outpatient Clinic, Gastroenterology Department, University Hospital Heraklion, Heraklion, Crete 70013, Greece

**Keywords:** Primary biliary cholangitis, quality of life, PBC-40

## Abstract

**Background:**

Primary biliary cholangitis (PBC) has been long associated with impairment of various aspects of health-related quality of life (HRQoL) with substantial differences among populations. This study evaluated for the first-time the HRQoL in Greek PBC patients in conjunction with clinical and laboratory parameters of patients.

**Methods:**

We analyzed prospectively collected data regarding the HRQoL by using the PBC-40 and SF-36 questionnaires in 374 Greek PBC patients and 131 age- and sex-matched non-PBC controls.

**Results:**

The PBC-40 questionnaire is a reliable tool for HRQoL assessment in Greek PBC patients (Cronbach's α > 0.7 for all domains). Implementation of PBC-40 and SF-36 demonstrated significant impairment of HRQoL in Greek PBC patients compared to controls (*P* < 0.001 for all comparisons). Emotional dysfunction, social impairment, and fatigue (100%, 80.5% and 78%, respectively) were amongst those with the highest, while cognitive dysfunction (32%) with the least impact on quality of life. Fatigue was associated with female sex (*P* = 0.02), longer disease duration (*P* = 0.01), presence of cirrhosis (*P* = 0.02) and positivity for PBC-specific ANA (*P* < 0.05), while social dysfunction with increased age (*P* < 0.001), longer disease duration (*P* < 0.001) and presence of cirrhosis (*P* = 0.004). Living in urban areas was linked to impaired social function (*P* = 0.04), cognition (*P* = 0.02), fatigue (*P* = 0.04) and increased total PBC-40 score (*P* = 0.01).

**Conclusions:**

Implementation of PBC-40 and SF-36 revealed impaired HRQoL in Greek PBC patients with fatigue, social and emotional dysfunction exerting the highest impact. However, total, and individual PBC-40 scores were lower than that reported in studies from Northern/Central Europe and Canada. Deranged HRQoL was associated with severity of liver disease and presence of PBC-specific ANA.

## Introduction

Primary biliary cholangitis (PBC) is an autoimmune-mediated liver disease which mainly affects middle-aged women. Although the disease might progress slowly without symptoms, it can affect patients’ life in numerous ways including progression to end-stage liver disease and complications related to liver failure.^[1]^

During the last decades there is an increasing appreciation that health-related quality of life (HRQoL) of PBC patients can be frequently impaired by various factors associated with the disease.^[2,3]^ Fatigue, itching, arthralgias, depression, sleep disturbances, social and emotional dysfunctioning may affect patients’ daily activity and wellbeing independently of the disease stage.

Assessment of HRQoL in PBC patients had previously been performed by using existing generic psychometric tools for the evaluation of HRQoL in other chronic diseases, though their specificity in evaluating PBC cases has been questioned.^[4,5]^ The need for a disease specific measure of HRQoL was satisfied by the PBC-40 questionnaire which was originally validated in UK PBC patients.^[6]^ It is a psychometric measure which consists of six domains of symptoms covering all factors associated with the disease that may affect HRQoL of PBC patients.

Application of PBC-40, along with its shorter versions PBC-27 and PBC-10 as well other measures for evaluation of several aspects of HRQoL, has highlighted impairment of HRQoL in PBC patients.^[6,7]^ Variations in the burden of HRQoL impairment have been reported across different populations pointing towards cultural inferences in PBC wellbeing.^[8]^ In line with this, our aim was to explore for the first-time aspects of HRQoL in PBC patients from Greece, originating from two distinct areas of the country.

## Materials and Methods

### Study design and subjects

We have prospectively enrolled 374 patients [336 (89.9%) females, mean age 60.4 ± 12.4 years] with well-defined PBC attending the outpatient clinic of two University Departments in Greece between 2017 and 2020. In detail, 300 patients were seen at the General University Hospital of Larissa (follow-up: 2000-2020) and 74 patients at the University Hospital of Heraklion, Crete, Greece (follow-up: 1991-2020). 131 sex- and age-matched healthy individuals from both centers were included as controls [110 (84%) females, mean age: 63.8 ± 16 years].

According to internationally accepted criteria, PBC diagnosis was established when at least two of the following criteria were present: a) positivity for AMA or anti-nuclear antibodies (ANA) specific for PBC, b) increased cholestatic enzymes for > 6 months and c) histological features of PBC.^[1]^

Patients having a PBC diagnosis of < 12 months, those suffering from other concurrent chronic liver diseases and patients post liver transplantation were excluded from the study. PBC patients with other co-morbidities that could have significantly influenced their quality of life, such as, diabetes mellitus with complications, heart failure ≥ NYHA II, renal failure requiring hemodialysis, dementia, psychosis, and malignancies were also excluded from the study. At last, patients of non-Greek origin or not comprehensive in Greek language were not included in the study.

Data related to patients’ demographics, lifestyle, residency, medical history, clinical and laboratory characteristics at the time of diagnosis as well as at the time of evaluation, stage of the disease, follow-up, duration, and response to treatment with ursodeoxycholic acid (UDCA) were collected.

In patients having undergone a liver biopsy at initial evaluation, classification was undertaken according to Ludwig’s classification.^[9]^ Otherwise, the diagnosis of cirrhosis was based on ultrasonography (presence of nodules or coarse echo pattern of the liver parenchyma along with irregular hepatic margins, length of spleen> 12 cm, portal vein diameter> 16 mm), and/or endoscopic findings of cirrhosis (varices, portal hypertensive gastropathy), and/or clinical findings of decompensation as we have reported.^[10]^ Data from liver elastography using Fibroscan^®^ 502 (Echosens, Paris, France) equipped with the standard M probe, used as a non-invasive evaluation of fibrosis near questionnaire completion, were also included where available. The Mayo Risk Score was used as a measure of disease severity.

AMA and PBC specific ANA positivity were also recorded. AMA were initially evaluated by indirect immunofluorescence on 5 μm fresh frozen sections of in-house rodent multiorgan tissue substrates, as described.^[11,12]^ AMA were also detected by enhanced performance M2 ELISA (M2 EP (MIT3) ELISA, QUANTA Lite (R), INOVA Diagnostics Inc. San Diego, CA, USA) and/or Western blot using in-house mitochondrial subfraction of rat livers.^[13]^ PBC-specific ANA against nuclear pore membrane glycoprotein 210 (anti-gp210) and nuclear sp100 protein (anti-sp100) were detected by commercial ELISAs according to the manufacturers’ instructions (INOVA Diagnostics).^[14]^

Evaluation of HRQoL in our study population was performed using a Greek version of the PBC-40, and the generic SF-36 questionnaires. Each patient received and completed both questionnaires by himself/herself at the same time under the supervision of an experienced hepatologist.

### Questionnaires

PBC-40: It consists of 40 items divided into the six domains of fatigue, itch, cognition, social function, emotional function and (other) symptoms. Each item might score from 1 (or 0 for itch and some items of social function and symptoms) to 5. Higher scores denote greater impact on HRQoL. The PBC-40 was translated to Greek and backward to English by two independent translators to ensure reliability and avoid discrepancies with the original English version.

SF-36 questionnaire: The SF-36 questionnaire is a generic, patient-reported measure of health status commonly used for the assessment of HRQoL in chronic diseases. It had already been translated and validated in Greek population.^[15,16]^ It consists of 36 items which are divided into the following 8 sections: physical functioning, role limitations due to physical health, role limitations due to emotional problems, vitality, mental health, social functioning, bodily pain, general health. Scale scores range between 0 and 100, with 100 representing the highest level of functioning possible.

### Ethics

The study was conducted in accordance with the protocol and the principles of the Declaration of Helsinki. The protocol was approved by the Ethics Committee of Thessaly University, Medical School. Each patient who participated in the study had been properly informed and gave his/her oral consent. Written consent was not required because the analysis used anonymous data that were obtained after each patient agreed to participate by codification of the subjects.^[17]^

### Statistical analysis

Analysis was performed using the software SPSS statistics 27.0 (IBM, Armonk, NY). Data distribution was assessed by Kolmogorov- Smirnov. Categorical variables were presented as proportions. Internal consistency of PBC-40 and SF-36 was measured by Cronbach’s coefficient α. A Cronbach’s alpha score of more than 0.7 was considered satisfactory.^[18]^ Bivariate analysis was performed by measuring correlations using Pearson’s correlation coefficient test (*r*). The ANOVA test was used to compare means of variables. Two-sided *P* values less than 0.05 were considered statistically significant.

## Results

### Demographic and clinical characteristics of PBC patients

Demographic and clinical characteristics of 374 patients at the time of HRQoL assessment are presented in Table 1. Patients from Crete were significantly older and had higher ALP, AST, total bilirubin and IgM levels and lower albumin compared to Larissa patients (Table 1). In addition, they had more frequently a liver biopsy during their initial evaluation compared to Larissa patients (*P* < 0.001). Amongst those having a liver biopsy, patients from Crete had more frequently advanced disease stage (*P* < 0.001). Moreover, patients from Crete had overall more severe disease in terms of fibrosis stage compared to patients from Larissa, as evaluated by liver elastography.

**Table 1:** Demographic and clinical characteristics of 374 Greek patients with PBC

Parameter	All patients (*n* = 374)	Patients from Crete (*n* = 74)	Patients from Larissa (*n* = 300)	*P* value
Gender (female)	337 (90.1)	66 (89.2)	271 (90.3)	NS
Age at questionnaire (years)	61.5 (25-87)	66 (42-87)	61 (25-85)	0.03
Residence (urban)	257 (68.7)	44 (59.5)	213 (71.0)	0.04
Unemployment^*^		NA	*n*=267	
			101 (36.8)	
Education		NA	*n*=211	
Primary			96 (45.5)	
Secondary			45 (21.3)	
Tertiary			70 (33.2%)	
Age at diagnosis (years)	52.9 ± 11.7	53.2 ± 10.8	52.9 ± 11.9	NS
Total follow up (months)	72 (0-331)	99 (0-331)	72 (0-252)	0.008
ALP (IU/L; UNL 120 IU/L)	94 (34-660)	113 (50-480)	92 (34-660)	0.02
γGT (IU/L; UNL 54 IU/L)	46.6 ± 60.6	56 ± 64	44.3 ± 60	NS
AST (IU/L; UNL 35 IU/L)	22 (7-127)	25 (11-120)	22 (7-127)	0.002
ALT (IU/L; UNL 35 IU/L)	27.2 ± 21	30 ± 24	26.6 ± 20	NS
Albumin (g/dL; 3.5-5.2 g/dL)	4.3 (0.5-5.6)	4.1 (2.8-4.8)	4.3 (3.1-5.6)	< 0.001
Total bilirubin (mg/dL; UNL 1.1mg/dL)	0.6 ± 0.5	0.7 ± 0.5	0.6 ± 0.4	NS
INR	1 (0.4-2.3)	1 (0.9-1.5)	1 (0.4-2.3)	0.01
IgM (mg/dL; UNL 200mg/dL)	*n* = 149 181 (23-2160)	*n* = 62 243 (45-2160)	*n* = 87 154 (23-1356)	0.007
Mayo Risk Score	1.3 ± 0.9	1.2 ± 0.9	1.4 ± 0.8	< 0.001
Biopsy^†^	165 (44.1)	46 (62.2)	119 (39.7)	< 0.001
Histopathological stage				
I-II	143 (86.6)	32 (69.6)	111 (93.3)	< 0.001
III-IV	22 (13.4)	14 (30.4)	8 (6.7)	< 0.001
AMA positive	359 (95.9)	71 (95.9)	288 (96.0)	NS
Sp100 positive	*n* = 295	*n* = 19	*n* = 276	
	39 (13.22)	2 (10.5)	37 (13.4)	NS
Gp210 positive	*n* = 296	*n* = 22	*n* = 275	
	35 (11.8)	5 (22.7)	31 (11.3)	NS
UDCA treatment	330 (88.2)	64 (86.5)	267 (89)	NS
UDCA treatment duration (months)	76.16 ± 36.34	102.8 ± 94	69.6 ± 67	NS
UDCA treatment response	304 (81.2)	56 (87.5)	248 (92.9)	NS
Bezafibrate treatment	5 (1.3)	0	5 (1.7%)	NS
Cirrhosis^‡^	38 (10.1)	17 (22.9)	21 (7.0)	< 0.001
Concomitant autoimmune disease ^§^	83 (22.2)	15 (20.27)	68 (22.7)	0.04
Fibroscan^¶^	206 (55.08)	51 (68.9)	155 (51.7)	0.002
Fibroscan score	7.8 ± 6.1	11.3 ± 9.8	6.7 ± 3.9	0.002
F0-F1	138 (67.0)	22 (43.1)	116 (74.8)	< 0.001
F2	33 (16.0)	6 (11.8)	27 (17.4)	< 0.001
F3	19 (9.2)	15 (29.4)	4 (2.6)	< 0.001
F4	16 (7.8)	8 (15.7%)	8 (5.2)	< 0.001

*There were no differences in the frequency of employed and unemployed PBC patients leaving in urban (65.6% and 34.4% respectively) and rural areas (54.4% and 45.7% respectively, *P* = 0.08). **^†^**Liver biopsy performed at first assessment; **^‡^**Cirrhosis at the time of questionnaire; §Concomitant autoimmune disease: Hashimoto’s thyroiditis in 41 (12.6%), Biermer’s anemia in 20 (5.3%), Sjogren syndrome in 8 (2.1%), Rheumatoid arthritis in 11 (2/9), Raynoud’s disease in 7 (1/9), inflammatory bowel disease in 6 (1/6), psoriatic arthritis in 5 (1/3), ankylosing spondylitis in 5 (1.3%), systemic lupus erythematosus in 4 (1.1%), vitiligo in 3 (0.8%); ^¶^Fibroscan performed in close proximity to questionnaire. NS, not statistically significant; NA, not applicable. Data are expressed as *n*(%) mean ± SD or median and range where appropriate. MWU, Man Whitney *U* test; χ^2^ test, chi square test with Yates correction. Abbreviations are the same as in the text.

### Reliability of scales

The internal consistency of the entire scale of PBC-40 and five of six domains was satisfactory as assessed by Cronbach’s coefficient α (α > 0.70). Estimates have been made for all patients as well as the two subgroups. The results were similar for all groups (Table 2).

**Table 2:** **Internal consistency of PBC-40 and its six domains as measured by Cronbach’s** α **(total PBC population and individual PBC cohorts)**

	All patients (*n* = 374)	Larissa (*n* = 300)	Crete (*n* = 74)
Symptoms	0.75	0.74	0.75
Itch	0.77	0.77	0.78
Fatigue	0.70	0.70	0.70
Cognition	0.74	0.73	0.74
Emotional	0.75	0.75	0.76
Social	0.71	0.71	0.70
PBC-40 scale	0.77	0.76	0.77

PBC: Primary biliary cholangitis.

The internal consistency of the entire scale of SF-36 questionnaire and its eight sections was good (Cronbach’ a score > 0.8) (Table 3).

**Table 3:** **Internal consistency of SF-36 and its eight sections as measured by Cronbach’s** α **(total PBC population and individual PBC cohorts)**

	All subjects (*n* = 374)	Larissa (*n* = 300)	Crete (*n* = 74)
Physical functioning	0.86	0.87	0.89
Role limitation due to physical health	0.83	0.86	0.87
Role limitation due to emotional problems	0.83	0.86	0.87
Vitality	0.85	0.87	0.87
Mental health	0.89	0.89	0.88
Social functioning	0.84	0.86	0.86
Pain	0.85	0.87	0.87
General health	0.85	0.88	0.87
SF-36	0.87	0.89	0.89

### HRQoL estimation

PBC-40 demonstrated impaired HRQoL in all Greek PBC patients compared to controls (*P* < 0.001 for comparisons of all individual components of PBC-40). Average total PBC-40 score was 77.8 ± 25.4. The scores of each PBC-40 domain for patients and controls are presented in Table 4. Patients from Crete had significantly higher mean scores for the total PBC-40 score and individual PBC domains except for the emotional domain compared to patients from Larissa (Supplementary Table 1).

**Table 4:** Results of PBC-40 in 374 Greek PBC patients and 131 controls

Domain	PBC patients	(*n* = 374)	Controls (*n* = 131)	*P* value
Symptoms	11 (1-26)		7 (6-19)	< 0.001
Itch	3 (0-30)		0	< 0.001
Fatigue	22 (3-53)		11 (10-16)	< 0.001
Cognition	9 (6-28)		6 (6-15)	< 0.001
Emotional	8 (3-27)		1 (1-4)	< 0.001
Social	19 (3-46)		8 (8-14)	< 0.001
PBC-40 scale	74 (8-151)		33 (31-56)	< 0.001

PBC: primary biliary cholangitis. MWU, Man Whitney *U* test.

Among 327 PBC patients, 49% (161/327) agreed their health to be rather excellent/good, while 51% (166/327) answered they disagree. Correspondingly, 92% (119/129) of healthy controls consider having excellent/good life and 8% (10/129) poor (*P* < 0.001). During the time of questionnaire completion, less than 20% of PBC patients reported having any of PBC-related symptoms (itching, dry mouth, dry eyes, fatigue, arthralgias and dysthymia).

As PBC-related symptoms occur also in non-PBC population, we further established clinical cut-offs indicative of significant impairment of individual components of PBC-40 in PBC patients based on data derived from our control population (> mean ± 2SD) (Table 5). In detail, 78% of PBC patients had significant fatigue, 80.5% social dysfunction, 100% emotional dysfunction, 32% cognition impairment and almost 50% had PBC-related symptoms. Overall, 87% of PBC patients had significantly higher PBC-40 total score compared to controls.

**Table 5:** Clinically significant cut offs in individual PBC domains and total PBC score based on mean ± 2SD for the control values

Symptoms domain	Control subject value		Clinical cut off	Positive in PBC population (*n* = 374%)	,	Positive in Larissa PBC population (*n* = 300, %)	Positive in Crete PBC population (*n* = 74, %)
Symptoms	7.45 ± 2.19		≥ 12	179 (48.0)		138 (46.0)	45 (61.0)
Fatigue	11.78 ± 1.49		≥ 15	292 (78.1)		231 (77.0)	61 (82.4)
Cognition	7.06 ± 2.10		≥ 12	121 (32.4)		81 (27.0)	40 (54.0)
Emotional	1.02 ± 0.26		≥ 2	374 (100.0)		300 (100.0)	74 (100.0)
Social	8.96 ± 1.95		≥ 13	301 (80.5)		238 (79.3)	63 (85.1)
PBC-40 scale	36.37 ± 6.64		≥ 50	327 (87.4)		260 (87.0)	67 (91.0)

Data presented as *n* (%) of PBC patients with clinically significant symptoms in PBC (total PBC population and individual PBC cohorts). PBC: primary biliary cholangitis.

Analysis of SF-36 revealed PBC patients to have significant impairment in all domains compared to healthy controls (*P* < 0.001 for all comparisons, Table 6). Among different domains, fatigue had the lowest score, while perception of general health was significantly impaired.

**Table 6:** Results of SF 36 domains in PBC patients and controls

SF36 domain	PBC (*n* = 374)	control (*n* =131)	*P* value
Physical functioning	80 (0-100)	100 (35-100)	< 0.001
Role limitations due to physical health-	100 (0-100)	100 (75-100)	< 0.001
Role Physical			
Role limitation due to emotional problems-	100 (0-100)	100	< 0.001
Role emotional			
Vitality	55 (0-100)	80 (45-80)	< 0.001
Mental Health	64 (4-100)	92 (64-92)	< 0.001
Social functioning	75 (0-100)	100 (55-100)	< 0.001
Bodily pain	87.5 (0-100)	100 (55-100)	< 0.001
General health	60 (0-125)	95 (60-95)	< 0.001

PBC: primary biliary cholangitis. MWU, Man Whitney *U* test.

### Correlations between scales

The convergent validity of the PBC-40 was estimated by calculated Pearson’ correlation between PBC-40 and SF-36 scores for all our groups. Significant correlations (*P* < 0.05) were seen between all domains of PBC-40 and SF-36 for the whole patients’ group (Table 7) and the two patients’ cohorts separately (Supplementary Tables 2 and 3).

**Table 7:** Pearson’s correlation between PBC-40 and SF-36 in all PBC patients (*n*=374)

	PF	RP	RE	VT	MH	SF	BP	GH
Symptoms	*r* = -0.309	*r* = -0.349	*r* = -0.346	*r* = -0.345	*r* = -0.444	*r* = -0.344	*r* = -0.441	*r* = -0.344
	*P* < 0.001	*P* < 0.001	*P* < 0.001	*P* < 0.001	*P* < 0.001	*P* < 0.001	*P* < 0.001	*P* < 0.001
Itch	*r* = -0.272	*r* = -0.227	*r* = -0.228	*r* = -0.250	*r* = -0.779	*r* = -0.301	*r* = -0.320	*r* = -0.271
	*P* < 0.001	*P* < 0.001	*P* < 0.001	*P* = 0.001	*P* < 0.001	*P* < 0.001	*P* < 0.001	*P* < 0.001
Fatigue	*r* = -0.420	*r* = -0.550	*r* = -0.528	*r* = -0.594	*r* = -0.156	*r* = -0.562	*r* = -0.584	*r* = -0.469
	*P* < 0.001	*P* < 0.001	*P* < 0.001	*P* < 0.001	*P* = 0.003	*P* < 0.001	*P* < 0.001	*P* < 0.001
Cognition	*r* = -0.302	*r* = -0.436	*r* = -0.452	*r* = -0.460	*r* = -0.121	*r* = -0.467	*r* = -0.407	*r* = -0.337
	*P* < 0.001	*P* < 0.001	*P* < 0.001	*P* < 0.001	*P* = 0.023	*P* < 0.001	*P* < 0.001	*P* < 0.001
Emotional	*r* = -0.347	*r* = -0.439	*r* = -0.448	*r* = - 0.572	*r* = -0.145	*r* = -0.603	*r* = -0.485	*r* = -0.574
	*P* < 0.001	*P* < 0.001	*P* < 0.001	*P* < 0.001	*P* < 0.001	*P* < 0.001	*P* < 0.001	*P* < 0.001
Social	*r* = -0.225	*r* = -0.322	*r* = -0.339	*r* =-0.497	*r* = -0.211	*r* = -0.507	*r* = -0.333	*r* = -0.523
	*P* < 0.001	*P* < 0.001	*P* < 0.001	*P* < 0.001	*P* < 0.001	*P* < 0.001	*P* < 0.001	*P* < 0.001

PF: physical functioning; RP: role limitation due to physical health; RE: role limitation due to emotional problem; VT: vitality; MH: mental health; SF: social functioning; BP: bodily pain; GH: general health.

### Correlations of PBC-40 with demographic and clinical features

Female PBC patients had significantly higher scores compared to males as far as domains fatigue (23.87 ± 10 *vs*. 19.65 ± 7.6, *P* = 0.01), symptoms (12.1 ± 4.6 *vs*. 9.2 ± 2.8, *P* < 0.001) and total PBC-40 score concerns (78.7 ± 25.7 *vs*. 68.5 ± 21.1, *P* = 0.02). Regarding SF-36 domains, female patients showed impaired mental health [62.5 (4100) *vs*. 68 (28-100), *P* = 0.04] and bodily pain [77.5 (0100) *vs*. 100 (33-100), *P* = 0.03) compared to males. On the contrary, there were no significant differences in any of the PBC-40 scores between male and female controls (Supplementary Table 4).

Except for female gender, total PBC-40 score correlated significantly with longer disease duration (*r* = 0.171, *P* = 0.001), presence of cirrhosis (*r* = 0.121, *P* = 0.02), the detection of anti-sp100 (*r* = 0.123, *P* = 0.02), living in urban areas (*r* = 0.133, *P* = 0.01) and unemployment (*r* =-0.161, *P* = 0.008). The domain symptoms correlated significantly with female gender (*r* = 0.191, *P* < 0.001) and domain itch with patients’ age (*r* = 0.139, *P* = 0.007) and INR (*r* = 0.114, *P* = 0.03). The domain fatigue correlated with female gender (*r* = 0.128, *P* = 0.01), longer disease duration (*r* = 0.107, *P* = 0.01), presence of cirrhosis (*r* = 0.123, *P* = 0.02), the detection of anti-sp100 (*r* = 0.132, *P* = 0.01) and anti-gp210 (*r* = 0.103, *P* = 0.04) and residency in urban areas (*r* = 0.107, *P* = 0.04). The domain cognition correlated significantly with longer disease duration (*r* = 0.136, *P* = 0.009), the presence of anti-sp100 (*r* = 0.166, *P* = 0.001) and anti-gp210 (*r* = 0.122, *P* = 0.02) and residency in urban areas (*r* = 0.119, *P* = 0.02). The domain social had statistically significant correlation with patients’ age (*r* = 0.174, *P* < 0.001), longer disease duration (*r* = 0.184, *P* < 0.001), presence of cirrhosis (*r* = 0.150, *P* = 0.004), unemployment (*r* = -0.227, *P* < 0.001) and residency in urban areas (*r* = 0.102, *P* = 0.04). PBC-40 scale or any of its domains had no correlation with AMA presence, Mayo risk score, UDCA treatment or response to UDCA.

There were significant differences across all three educational levels (primary, secondary and tertiary education) regarding domains itch, social, emotional and total PBC-40 score. Compared to patients with higher education, those with primary education has significantly higher scores for itch [3 (0-9) *vs*. 3 (0-30), *P* < 0.001], social [16 (6-33) *vs*. 21 (4-39), *P* < 0.001], emotional domains [6 (3-15) *vs*. 9.5 (3-15), *P* = 0.005] and total PBC-40 score [68.5 (37-128) *vs*. 78.5 (40-126], *P* = 0.008).

Patients with significant fatigue (≥ 15, *n* = 292) had significantly higher scores in all PBC-40 domains, including the total PBC-40 score compared to PBC patients with no clinically significant fatigue (< 15, *n* = 82; Supplementary Table 5). Comparison of 82 patients [75 females (91%), mean age: 57.5 ± 12.5 years, 6 cirrhotic, 74 (90%) on UDCA treatment and 71 (96%) with response to treatment] experiencing no itch at the time of questionnaire completion, with those reporting itching revealed that patients without itch had significantly less impaired HRQoL scores except for cognition (Supplementary Table 6). Multivariate analysis revealed better HRQoL in patients with no itch to significantly correlate with younger age (*P* = 0.02) and lower bilirubin levels (*P* = 0.04).

## Discussion

In this study we set out to explore the performance of PBC-40 as a tool for assessing HRQoL in Greek PBC patients. As recently highlighted in a systemic review, amongst methods to assess patient reported outcomes in PBC, PBC-40 is so far, the most validated.^[19]^ Moreover, we used the SF-36 questionnaire, which has been previously validated in the Greek population, and correlated its findings with PBC-40.^[15]^

We demonstrated PBC-40 to be a reliable tool for the assessment of HRQoL in Greek PBC patients, as the overall internal consistency of the score was high with Cronbach’s α > 0.7 for all domains. Analogously, the overall internal consistency of SF-36 and its eight sections was also high (Cronbach’s α > 0.8), which is in line with previous evaluation of this questionnaire in the Greek general population.^[15]^ As reported in previous studies, there were striking correlations between individual components of PBC-40 and SF-36, which underscores the convergent validity of the former measure.^[20]^

Next, implementation of both tools proved HRQoL to be impaired in Greek PBC patients compared to controls. Concerning the PBC-40 score, our data are consistent with those from several studies showcasing impaired HRQoL in PBC patients around the world.^[3,8,20,21]^ However, of interest, Greek PBC patients had significantly lower total and individual PBC-40 scores compared to other PBC populations mainly from North or Central Europe and Canada.^[3,21,22]^ Of note, our non-PBC controls had also lower scores compared to the control populations included in other studies,^[3]^ that lends further support to the assumption that HRQoL is influenced by an interplay between cultural, social, financial and geographical factors.^[2,8]^

Our study assessed HRQoL in PBC patients from Greece by prospectively recruiting PBC patients from two well-established liver centers in Greece. Data from both centers have previously shown PBC prevalence to be among the highest in Europe.^[23,24]^ We anticipate PBC patients in our study to be representative of the general PBC population in Greece, as both centers act also as primary and secondary healthcare facilities based on the structure of the existing healthcare system in our country. This is of great importance as previous work from the UK has emphasized that evaluation of clinic-based PBC patients visiting tertiary centers might lead to overestimation of symptoms such as fatigue when comparing them to community based PBC population.^[25]^Moreover, our population is considered homogeneous, as most of the PBC patients were Caucasians of Greek origin.^[23,24]^ This is of outmost importance as differences in socioeconomic, cultural, even geographical characteristics can have an impact on HRQoL in PBC patients and thus may lead to potential bias in cases of studies of multi-ethnic origin.^[2]^ Along this line, a study encompassing patients from the UK, Spain, Italy, and Japan have shown significant differences in fatigue, cognitive and emotional domains amongst the four countries, suggesting that differences in latitude, genetics or even cultural characteristics could play a decisive role in HRQoL in PBC patients.^[8]^ However, in our study we believe that the reported differences between the two PBC cohorts were mainly attributed to the more advanced disease of the Cretan cohort at the time of questionnaire completion compared to the Larissa group. The latter was also confirmed histologically, as liver biopsy was performed more frequently in patients from Crete because histology has been a prerequisite for establishing definite PBC diagnosis during the previous decades.

Our study manifested significant differences between perceived HRQoL and presence of symptoms reported by PBC patients. While up to 50% of PBC patients regarded their well-being to be good or excellent, implementation of PBC-40 showed significant impairment in most of our cohort (>80%). Emotional dysfunction, social impairment and fatigue were amongst those with the highest impact on quality of life. Fatigue has been identified as the most frequent and disabling symptom in PBC patients, with a study demonstrating stable characteristics over time.^[26]^ Accordingly, we have demonstrated that PBC patients with significant fatigue have higher scores of all other PBC-40 domains, verifying previous data which suggested that there is interrelationship between individual components of PBC-40.^[3,27]^

Female PBC patients had worse fatigue scores, and this is line with previous reports from the UK and Japan.^[28,29]^ Differences in fatigue scores between genders were attributed to autonomic dysfunction in females in a study from the UK.^[28]^ We did not evaluate PBC patients for symptoms of autonomic dysfunction, as our goal was mainly to evaluate the feasibility of PBC-40 in a well-defined cohort of Greek PBC patients. Fatigue was worse in patients with cirrhosis in our study, which failed to agree with the majority of studies displaying fatigue and HRQoL in general to be independent of disease severity.^[2,3,20]^ In line with this, presence of PBC-specific ANA, namely anti-sp100 and anti-gp210, known to characterize patients with unfavorable disease outcome, including more severe histological stage, were associated with worse fatigue and cognitive function in our population.^[11,14]^ However, contrary to our findings, fatigue was not associated with cirrhosis in the Polish PBC population.^[20]^

Worse social domain scores were linked to increased age, longer duration of follow-up and cirrhosis, all indicative of severe, long-standing disease. Our data do not comply with those from other studies showcasing presentation of PBC at younger age to be associated with worse HRQoL with social functioning exerting the greatest contribution to this impairment.^[27]^ It is of interest that cognitive function was the least impaired domain in our population. Previous reports have emphasized significant differences in cognitive impairment between different PBC populations.^[30,31]^ Whether cognitive impairment might relate to individual predisposing factors prevalent in specific areas or whether such patients constitute a separate group with distinct features needs to be further evaluated.^[32]^

Akin to previous studies, itch had the lowest score among PBC-40 domains.^[3,22]^ This might probably relate to the fact that most of our patients were under treatment with UDCA that is shown to partially ameliorate the intensity of itching. Living in urban compared to rural areas has been one of the features associated with impairment in several aspects of HRQoL in our population, including fatigue, cognition, social and the total PBC-40 score. The impact of residency on HRQoL has been linked to various factors, like access to health care facilities and information, differences in income and educational status as well as ethnicity, sex, and disease burden. Our data are in line with a study assessing HRQoL in the general population of Thessaly (place of residency of most of our patient population), where rural residency was associated with better general health in individuals of Greek origin.^[33]^ However, whether living in the Greek countryside offers advantages needs to be evaluated in largescale studies. Even though data on patients’ income were not available, unemployment and lower educational attainment were factors significantly affecting domains such as social, emotional and the total PBC-40 score in our PBC patients. Our data are in line with the study by Parikh-Patel and colleagues showing lower level of education to be significantly associated with lower levels of functional capabilities in PBC patients from the US.^[34]^ Moreover, several studies illustrate low socioeconomic status, as attested by several interrelated factors such as lower income, lower educational level and impaired social support, to affect HRQoL in various autoimmune diseases.^[35]^

Our study has some limitations. Unfortunately, data on other covariates which can potentially affect quality of life, such as income and social support were not included as per protocol. In addition, data on education and employment were available only in Larissa group which, however, was the largest group of patients in the study. Still, we report for the first-time results based on prospectively collected questionnaires in a large number of well-characterized Greek PBC patients from two geographical regions of the country with long-term follow-up.

In conclusion, we have demonstrated that PBC-40 is a reliable tool for HRQoL assessment in Greek PBC patients. Both PBC-40 and SF-36 revealed significant impairment of HRQoL in a representative sample of PBC patients from two geographical areas of the country, compared to healthy controls. However, the total and individual PBC-40 scores were lower both in patients and controls compared to scores in PBC and control populations from Europe and Canada, while cognitive function was the least impaired domain in our population suggesting that HRQoL might be influenced by many local factors. Emotional dysfunction, social impairment and fatigue displayed the highest impact on quality of life in our patients while female gender, cirrhosis, and presence of PBC-specific ANA played a decisive role in impairing individual components of HQRoL.

## Supplementary Material

Supplementary material
