# Supplementary material for: Total and individual PBC-40 scores are reliable for the assessment of health-related quality of life in Greek patients with primary biliary cholangitis
Source: J Transl Int Med. 2023 Sep 2;11(3):246–54. doi: 10.2478/jtim-2023-0098 (PMC10561069; doi:10.2478/jtim-2023-0098)
Supplement: Supplementary file 1 — Supplementary material [file jtim-2023-0098_sm.pdf]

Supplementary Material

Supplementary Table 1. PBC-40 in PBC patients from Larissa and Crete

Domain	Larissa (n = 300)	Crete (n = 74)	P value
Symptoms	11 (1-26)	13 (5-26)	<0.001
Itch	3 (0-30)	44.5 (0-13)	0.001
Fatigue	22 (11-49)	27.5 (13-53)	<0.001
Cognition	7 (6-28)	12 (6-27)	<0.001
Emotional	8.27 ± 4.09	9 ± 3.48	0.06
Social	19 (3-46)	21 (7-43)	0.04
Total PBC-40 score	71 (8-145)	89 (32-151)	<0.001

PBC: primary biliary cholangitis.

Supplementary Table 2. Pearson's correlation between PBC-40 and SF-36 in PBC patients from Larissa

	PF	RP	RE	VT	MH	SF	BP	GH
Symptoms	r=-0.290	r=-0.389	r=-0.457	r=-0.441	r=-0.215	r=-0.424	r=-0.460	r=-0.285
	P=0.013	P<0.001	P<0.001	P<0.001	P=0.041	P<0.001	P<0,001	P=0.015
Itch	r=-0.303	r=-0.185	r=-0.278	r=-0.261	r=-0.257	r=-0.279	r=-0.270	r=-0.279
	P=0.030	P<0.001	P=0.041	P=0.035	P=0.031	P=0.023	P=0.023	P=0.030
Fatigue	r=-0.490	r=-0.500	r=-0.478	r=-0.689	r=-0.495	r=-0.538	r=-0.583	r=-0.571
	P<0.001	P<0.001	P<0.001	P<0.001	P<0.001	P<0.001	P<0.001	P<0.001
Cognition	r=-0.375	r=-0.468	r=-0.525	r=-0.402	r=-0.444	r=-0.596	r=-0.473	r=-0.583
	P<0.001	P<0.001	P<0.001	P<0.001	P<0.001	P<0.001	P<0.001	P<0.001
Emotional	r=-0.317	r=-0.403	r=-0.480	r=-0.273	r=-0.348	r=-0.507	r=-0.445	r=-0.351
	P=0.007	P<0.001	P<0.001	P<0.001	P<0.001	P<0.001	P<0.001	P=0.002
Social	r=-0.358	r=-0.432	r=-0.434	r=-0.569	r=-0.582	r=-0.596	r=-0.362	r=-0.728
	P=0.002	P<0.001	P<0.001	P<0.001	P<0.001	P<0.001	P= <0.001	P<0.001

PBC: primary biliary cholangitis; PF: physical functioning; RP: role limitation due to physical health; RE: role limitation due to emotional problem; VT: vitality; MH: mental health; SF: social functioning; BP: bodily pain; GH: general health.

Supplementary Table 3. Pearson's correlation between PBC-40 and SF-36 in PBC patients from Crete

	PF	RP	RE	VT	MH	SF	BP	GH
Symptoms	r=-0.290	r=-0.389	r=-0.457	r=-0.441	r=-0.215	r=-0.424	r=-0.460	r=-0.285
	P<0.001	P<0.001	P<0.001	P<0.001	P<0.001	P<0.001	P<0,001	P=0.015
Itch	r=-0.185	r=-0.178	r=-0.261	r=-0.257	r=-0.279	r=-0.279	r=-0.270	r=-0.259
	P=0.022	P=0.014	P=0.014	P=0.031	P=0.018	P=0.023	P=0.023	P=0.022
Fatigue	r=-0.490	r=-0.500	r=-0.478	r=-0.689	r=-0.495	r=-0.538	r=-0.583	r=-0.571
	P<0.001	P<0.001	P<0.001	P<0.001	P<0.001	P<0.001	P<0.001	P<0.001
Cognition	r=-0.375	r=-0.468	r=-0.525	r=-0.402	r=-0.444	r=-0.507	r=-0.445	r=-0.351
	P<0.001	P<0.001	P<0.001	P<0.001	P<0.001	P<0.001	P<0.001	P=0.002
Emotional	r=-0.316	r=-0.403	r=-0.434	r=-0.569	r=-0.582	r=-0.596	r=-0.362	r=-0.728
	P<0.001	P<0.001	P<0.001	P<0.001	P<0.001	P<0.001	P= <0.001	P=0.002
Social	r=-0.583	r=-0.473	r=-0.596	r=-0.548	r=-0.573	r=-0.480	r=-0.432	r=-0.358
	P<0.001	P<0.001	P<0.001	P<0.001	P<0.001	P<0.001	P<0.001	P<0.001

PBC: primary biliary cholangitis; PF: physical functioning; RP: role limitation due to physical health; RE: role limitation due to emotional problem; VT: vitality; MH: mental health; SF: social functioning; BP: bodily pain; GH: general health.

Supplementary Table 4. Comparison of PBC-40 scores between healthy females and males.

PBC 40 domain	Female (n = 110)	Male (n = 21)	P value
Symptoms	7.30 ± 2.17	7.76 ± 2.23	0.27
Fatigue	11.71 ± 1.37	11.93 ± 1.73	0.43
Cognition	7.00 ± 2.16	7.19 ± 2.09	0.63
Social	8.87 ± 1.89	9.14 ± 2.07	0.47
Emotional	1.03 ± 0.32	1.00 ± 0.00	0.49
Total PBC	36.09 ± 6.64	36.95 ± 6.68	0.49

PBC: Primary biliary cholangitis.

Supplementary Table 5. Comparison of PBC-40 scores between PBC patients with and without fatigue

	No fatigue (< 15)	Fatigue (≥ 15)	P value
Other symptoms	9.16 ± 3.2	12.53 ± 4.6	< 0.001
Itch	2.32 ± 4.8	4.13 ± 3.3	< 0.001
Cognitive	6.72 ± 1.5	11.25 ± 5.1	< 0.001
Social	14.6 ± 5	21.64 ± 8.2	< 0.001
Emotional	6.43 ± 4.2	8.96 ± 3.8	< 0.001
Total PBC-40	51.46 ± 9.3	85.08 ± 23.8	< 0.001

PBC: Primary biliary cholangitis.

Supplementary Table 6. Comparison of PBC-40 scores between PBC patients with and without itching

	No itch (0)	Itch (≥ 1)	P value
Other symptoms	9 (1-26)	12 (5-26)	0.030
Fatigue	18 (11-48)	23 (3-53)	0.001
Cognitive	7 (6-24)	9 (6-28)	0.200
Social	16 (3-37)	20 (6-46)	< 0.001
Emotional	6 (2-23)	9 (3-27)	< 0.001
Total PBC-40	61 (8-128)	79 (30-151)	< 0.001

PBC: Primary biliary cholangitis.
